# Toxigenic *Vibrio cholerae* strains in South-East Queensland, Australian river waterways

**DOI:** 10.1128/aem.00472-23

**Published:** 2023-10-06

**Authors:** Murari Bhandari, Irani U. Rathnayake, Lawrence Ariotti, Brett Heron, Flavia Huygens, Mitchelle Sullivan, Amy V. Jennison

**Affiliations:** 1 Centre for Immunology and Infection Control, Queensland University of Technology, Brisbane, Queensland, Australia; 2 Department of Health, Public Health Microbiology, Forensic and Scientific Services, Brisbane, Queensland, Australia; University of Delaware, Lewes, Delaware, USA

**Keywords:** *vibrio cholerae*, toxigenic strains, AMR, virulence factors, mobile genetic elements, polymerase chain reaction

## Abstract

Cholera is a major public health problem in developing and underdeveloped countries; however, it remains of concern to developed countries such as Australia as international travel-related or locally acquired cholera or diarrheal disease cases are still reported. Cholera is mainly caused by cholera toxin (CT) producing toxigenic O1 and O139 serogroup *Vibrio cholerae* strains. While most toxigenic *V. cholerae* cases in Australia are thought to be caused by international-acquired infections, Australia has its own indigenous toxigenic and non-toxigenic O1 and non-O1, non-O139 *V. cholerae* (NOVC) strains. In Australia, in the 1970s and again in 2012, it was reported that south-east Queensland riverways were a reservoir for toxigenic *V. cholerae* strains that were linked to local cases. Further surveillance on environmental reservoirs, such as riverways, has not been reported in the literature in the last 10 years. Here we present data from sites previously related to outbreaks and surveillance sampling to detect the presence of *V. cholerae* using PCR in conjunction with MALDI-TOF and whole-genome sequencing. In this study, we were able to detect NOVC at all 10 sites with all sites having toxigenic non-O1, non-O139 strains. Among 133 NOVC isolates, 22 were whole-genome sequenced and compared with previously sequenced Australian O1 and NOVC strains. None of the samples tested grew toxigenic or non-toxigenic O1 or O139, responsible for epidemic disease. Since NOVC can be pathogenic, continuous surveillance is required to assist in theclinical and envir rapid identification of sources of any outbreaks and to assist public health authorities in implementing control measures.

**IMPORTANCE:**

*Vibrio cholerae* is a natural inhabitant of aquatic environments, both freshwater and seawater, in addition to its clinical significance as a causative agent of acute diarrhea and extraintestinal infections. Previously, both toxigenic and non-toxigenic, clinical, and environmental *V. cholerae* strains have been reported in Queensland, Australia. This study aimed to characterize recent surveillance of environmental NOVC strains isolated from Queensland River waterways to understand their virulence, antimicrobial resistance profile and to place genetic current *V. cholerae* strains from Australia in context with international strains. The findings from this study suggest the presence of unique toxigenic *V. cholerae* in Queensland river water systems that are of public health concern. Therefore, ongoing monitoring and genomic characterization of *V. cholerae* strains from the Queensland environment is important and would assist public health departments to track the source of cholera infection early and implement prevention strategies for future outbreaks. The genomics of environmental *V. cholerae* could assist us to understand the natural ecology and evolution of this bacterium in natural environments with respect to global warming and climate change.

## INTRODUCTION

*Vibrio cholerae* is the causative agent of severe disease, cholera that can be endemic and epidemic disease leading to pandemics. Mainly, O1 and O139, two serogroup *V. cholerae* strains, are responsible for major outbreaks and pandemics. To date, seven pandemics have been reported. The first six pandemics are thought to be caused by the classical biotype of *V. cholerae* O1 strains, whereas the current pandemic is caused by El Tor strains. The facultative human pathogen *V. cholerae* is also a habitat of estuarine and brackish or saltwater making the environment a reservoir of *V. cholerae*.

Cholera is caused by the ingestion of pathogenic *V. cholerae* (10^2^–10^6^ colony-forming units), which colonize the small intestine, multiply, secrete cholera toxin (CT), and are released into the environment via fecal contamination and dissemination into different locations via other means (1). Pathogenic O1 and O139 serogroup *V. cholerae* strains, which typically cause outbreaks and epidemics, produce CT and the colonization factor, toxin-coregulated pilus (TCP). By contrast, more than 95% of non-O1 and non-O139 serogroup *V. cholerae* lack these major virulence factors (2, 3). However, non-O1 and non-O139 strains contain other accessory virulence and regulatory genes, as mentioned previously, which may contribute directly or in a synergistic way to the infection process leading to diarrheal illness (4–11). Several recent studies have shown that these virulence genes are also distributed among diverse serogroups that constitute the environmental reservoir (3, 12, 13). It is believed that these environmental strains are precursors of pathogenic strains; therefore, monitoring environmental reservoirs for the presence of *V. cholerae* strains with pathogenic potential is likely to assist source identification in cholera outbreaks or sporadic cases of gastroenteritis.

During different cholera epidemics, isolation of the *V. cholerae* bacterium is correlated with environmental sources under favorable conditions with similar virulence properties and pathogenic potential (14, 15). However, detection and typing of the bacterium from an environmental source can prove challenging. Normally, the occurrence of *V. cholerae* in the aquatic environment can be in two states: in a viable and culturable state (VAC) and/or in a viable but not culturable physiological (VBNC) state, and therefore can be missed using conventional (culture based) microbiological methods (16). In addition, *V. cholerae* cells might be present in an environmental source at very low abundance within complex microbial communities and this may also hinder their detection by PCR or shotgun metagenomic sequencing. All these issues limit our capability to track the source of cholera outbreaks. Thus, improvements in isolation and detection techniques are crucial for investigating cholera outbreaks and to understanding the role of environmental reservoirs of toxigenic *V. cholerae* strains or their genes and antimicrobial properties. A genomic approach using whole-genome enrichment and next-generation sequencing for direct genotyping and metagenomic analysis of low abundant *V. cholerae* from natural water collected from the Morogoro River provided insights into virulence genes and the identification of *V. cholerae* (17); however, costs are prohibitive for broad surveillance. Rather, a dual PCR and next-generation sequencing approach on targeted strains would minimize the cost, provide high resolution of genome data and also be feasible for regular surveillance of the environment.

Globally, dynamic *V. cholerae* strains, their serological switching, and disease occurrence with respect to climate change are fascinating and are of public health concern (18). Australia has its own local toxigenic and non-toxigenic O1 and NOVC strains which have been isolated from clinical and environmental sources with pathogenic potential and with diverse antibiotic resistance profiles (3, 19). This has warranted further surveillance of Queensland river water systems. Our study elucidated the presence of unique toxigenic and *int*SXT-containing *V. cholerae* isolates prevalent in Queensland river water systems using PCR and whole-genome sequencing (WGS) methods.

## MATERIALS AND METHODS

### Sample collection sites

#### Study area, water sample collection, and processing

Water samples from Albert and Logan Rivers in South-East Queensland, Australia were collected in February and March 2021 from 10 sites. Some of these sites are being used by the local population for recreational activities (swimming) and were spatially associated with previous outbreak cases, as well as a previous *V. cholerae* surveillance project. These sites are located about 40–90 km from Brisbane. All samples were collected in triplicate using aseptic techniques in sterile bottles (TechnoPlas) placed in a cooler box and transported at ambient temperature from the site of collection to the Forensic and Scientific Services laboratory, Coopers Plains, Brisbane. All samples were processed the same day within 3–4 hours from sample collection. Collected samples were also tested for pH and salinity levels. Each sample was collected in 1 L volumes and triplicate and concentrated by filtration through a 0.45-µM bacteriological membrane filter (Millipore).

### Methods used to detect *V. cholerae* in water samples

#### Enrichment method and plating

Collected and concentrated samples were enriched in alkaline peptone water (APW) at 37°C for 6 hours and 1 mL culture was inoculated in fresh 10 mL APW broth and incubated at 37°C overnight. The following day, approximately 5 µL from the 10-mL enriched APW broth (a loop full) was streaked onto thiosulfate-citrate-bile salts-sucrose (Elken, Tokyo, Japan) agar and incubated at 37°C for 18 hours. Colonies with the characteristic appearance (typical yellow colonies) were sub-cultured onto Horse blood agar (HBA) and incubated overnight at 37°C. These cultures were used for *V. cholerae* identification using MALDI-TOF (Bruker). MALDI-TOF confirmed cultures were processed further for PCR characterization

#### Characterization of isolates using PCR

A multiplex PCR test for the amplification of the *V. cholerae* species-specific gene, *hlyA* (encoding hemolysin)*,* present in almost all classical, El Tor, and non-O1 strains, was carried out as described in previous studies (20–23). The genes responsible for O-antigen biosynthesis and for serotype-specific determinants are located in the *rfb* region of the *V. cholerae* genome. The *rfb* genes are specific for *V. cholerae* O1 and O139, the *ctxA*, *ctxAB* encoding subunit A and, A & B of cholera toxin, and *hlyA* were amplified using a penta-plex PCR. Initially, 1 mL of broth was centrifuged at 13,000 *g* to collect cell pellets and resuspended in 400 µL of TE buffer containing 1 mM EDTA disodium salt was boiled for 10 min to extract DNA. Primer sequences for the target genes are outlined in Table 1. DNA samples (2 µL) were added to the PCR penta-plex mix for a total volume of 25 µL containing 12.5 µL Qiagen Multiplex Mastermix, 1 µL of reverse and forward primer sets for each of the target genes (O1 *rfb*, O139 *rfb*, *ctxA*, *ctxAB*, and *hlyA*), and 0.5 µL of sterile water.

**TABLE 1:** PCR primers used in this study

Primer name	Primer sequence	PCR amplicon size (bp)	Reference
O1 *rfb-*F	5-GTTTCACTGAACAGATGGG-3	192	(24)
O1 *rfb-*R	5-GGTCATCTGTAAGTACAAC-3	192	
O139 *rfb-*F	5-AGCCTCTTTATTACGGGTGG-3	449	(24)
O139 *rfb-*R	5-GTCAAACCCGATCGTAAAGG-3	449	
VCT-F	5-ACAGAGTGAGTACTTTGACC-3	308	(24)
VCT-R	5-ATACCATCCATATATTTGGGAG-3	308	
*ctxAB-*F	5-AGGTGTAAAATTCCTTGACGA-3	385	(25)
*ctxAB-*R	5-TCCTCAGGGTATCCTTCATC-3	385	
*hlyA-*F	5-GCAAACAGCGAAACAAATACC-3	497	(25)
*hlyA-*R	5-TCCACCCCACCAGTCACC-3	497	

Amplification conditions used for PCR were one cycle of 15 min at 95°C for initial denaturation of DNA, followed by 30 s at 95°C, 1 min at 60°C and 72°C for 35 cycles with a final extension for 7 min at 72°C. After amplification, 10 µL of each PCR product was examined by electrophoresis in a 2% agarose gel containing ethidium bromide (5 µL/100 mL agarose). The gel containing the amplified DNA was viewed using the Gel Doc imaging system (Bio-Rad).

#### Sequencing of selected *V. cholerae* strains

**Fig 1:**
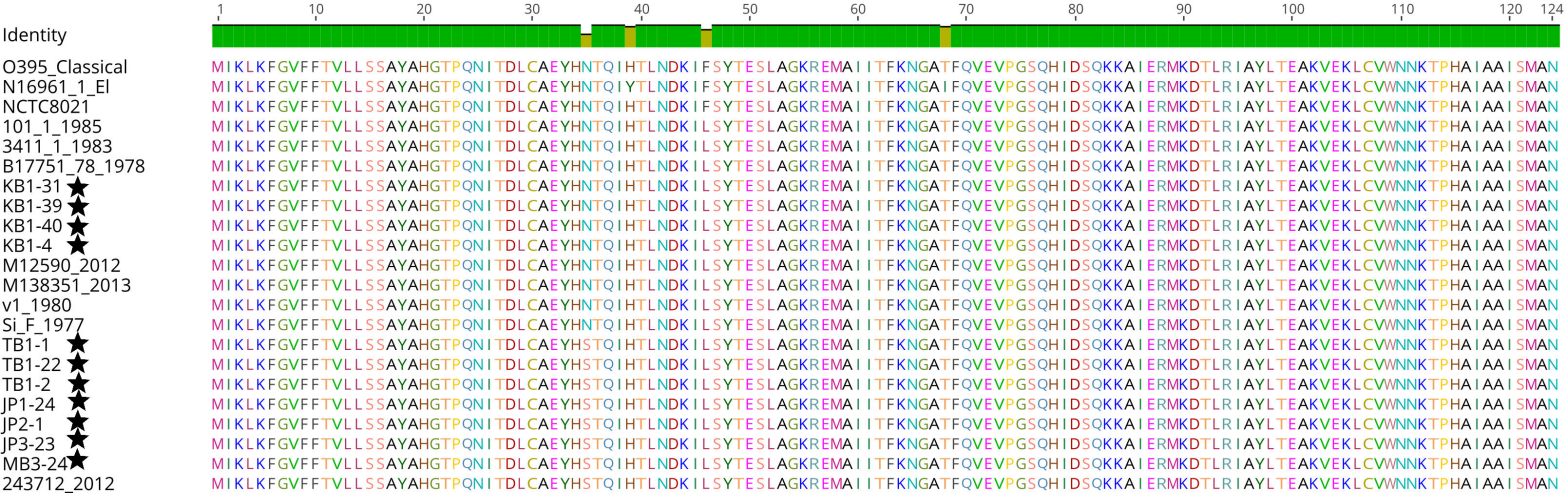
Multiple sequence alignment of ctxB amino acid sequences from this study and previous O1, NOVC isolates compared with seventh pandemic El Tor and sixth pandemic classical strains. Strains sequenced in this study are noted with an asterisk (*).

All the cholera toxin-positive strains and 10 cholera toxin-negative NOVC strains from each site (*n* = 133 in total) were stored as glycerol stocks for further analysis. Further genetic characterization was performed by WGS on selected strains as outlined in Data set 1. *V. cholerae* non-O1, non-O139 strains isolated from environmental samples (*n* = 22) were sequenced and their sources, locations of isolation, biotypes, sequence types, and year of isolation are outlined in Fig. 1; Data set 1. The WGS protocol was followed as per our previous studies (3, 19). Briefly, DNA was extracted from isolates grown overnight at 37°C on HBA (Edwards Group Holdings, Australia), and using the QiaSymphony DSP DNA Mini kit (Qiagen) according to the manufacturer’s protocol. DNA was prepared for sequencing using the Nextera XT kit (Illumina) and sequenced on the NextSeq500 using the NextSeq 500 Mid Output v2 kit (300 cycles) (Illumina) according to the manufacturer’s instructions. Sequence reads for the *V. cholerae* isolates were trimmed with Trimmomatic v0.36 (26) and quality checked by FastQC v0.11.5 and MultiQC v1.1 (27). Sequence reads with >75% of the read length in the green zone of the mean quality scores graph on FastQC (>Q28), have an average read length of >120 bp and gave the majority of reads over 140 bp according to the sequence length distribution graph were selected. *De novo* assemblies were generated with the SPAdes assembler v3.12.0 (28); the quality of the assemblies was analyzed using QUAST 4.6.3; and annotation was performed using Prokka. The quality of assemblies was determined based on the contigs ≥ 500 bp in length, which must be less than 500 in number and the total length of the assembled contigs should be similar (within 30%) to the expected genome median from National Center for Biotechnology Information (NCBI) genomes.

### Single nucleotide polymorphism-based phylogenetic analysis

To perform comparative phylogenetic analysis among recently sequenced 22 strains with other 83 publicly available *V. cholerae* strains from Australia and overseas (*n* = 105 in total), *V. cholerae* genome sequences were obtained from the public database (NCBI- https://www.ncbi.nlm.nih.gov) as shown in Data set 1. Core single nucleotide polymorphism (SNPs) were determined using Snippy version 4.3.6 (https://github.com/tseemann/snippy) using the seventh pandemic *V. cholerae* O1 El Tor N16961 genome as a reference (GenBank accession number NZ_CP028827.1 and NZ_CP028828.1). Core SNPs were aligned and used to generate a maximum likelihood tree using fasttree v2.1.10. The distance matrix was constructed with snp-dists v0.6.2. Interactive Tree of Life (iTol) v5.6 was used for the visualization of the phylogenetic tree (https;//itol.embl.de/) (29).

### Genotyping, virulence, and antimicrobial resistance gene analysis of *V. cholerae*

Identification of all the *V. cholerae* strains selected for this study was confirmed using a combination of MALDI-TOF and PCR. Furthermore, WGS of all the strains were characterized by Cholera Finder (https://cge.food.dtu.dk/services/CholeraeFinder/) in the Center for Genomic Epidemiology web server (https://cge.food.dtu.dk/services/CholeraeFinder/)—online tool CholeraFinder v1.0. This tool uses BLAST as a search engine and was used to determine the presence of the species-specific gene (ompW), serogroup-specific genes (*rfbV*-O1, *wbfZ*-O139), biotype-specific genes (*ctxB, rstR, tcpA*), seventh pandemic-specific gene (VC2346), collective virulence genes (*ctxA*, *ctxB*, *zot*, *ace*, *tcpA*, *hlyA*, *stn*, *chxA*, *rtxA*, *ompU*, *toxR*, *mshA*, *makA*, *als*, T3SS, T6SS)*,* pathogenic islands (VPI-1, VPI-2, VSP-I, and VSP-II) in all *V. cholerae* strains with a threshold equal to 95% identity and 60% coverage as previously described (30). For *ctxB* genotyping, the *ctxB* gene sequences were extracted from annotated strains and analyzed in Geneious (https://www.geneious.com/) using a multiple sequence alignment tool (CUSTALW), (http://www.clustal.org/clustal2/). This was used to determine sequence similarities and uniqueness among ctxB and ctxA amino acid sequences containing Australian *V. cholerae* strains.

Antimicrobial resistance genes, mobile genetic element (SXT), plasmids, and class 1 integron genes were detected using the Resfinder tool (https://cge.food.dtu.dk/services/ResFinder/) for all *V. cholerae* strains with a threshold equal to 95% identity and 60% coverage as previously described (30).

## RESULTS

Of the 10 sample sites, all sites had NOVC strains including toxigenic *V. cholerae* (Table 2). For both sample collection rounds, an increase in water temperature and a higher population of *V. cholerae* strains were detected based on the PCR amplicon intensity compared to lower temperature sites. Overall, the pH of the Albert and Logan Rivers was ~7, and a salinity was between 0.09 and 0.199 PSU (Table 2). Interestingly, no toxigenic *V. cholerae* strains were detected at Kerry Bridge which had the lowest temperature, pH, and salinity in the first round of collection; however, toxigenic *V. cholerae* strains were isolated in the second round of collection without significant differences in temperature, pH, and salinity. Also, two sites of the Logan River (South Macleans Bridge and Wendt Park Logan Village) were negative for toxigenic *V. cholerae* in the second round of sample collection.

**TABLE 2:** Location of sample collection sites and physical parameters measured during sample collection

			February					March				
SN^*a*^	L^*b*^	R^*c*^	WT^*d*^	pH	C^*e*^	S^*f*^	± TV^*g*^	WT^*d*^	pH	C^*e*^	S^*f*^	± TV^*g*^
1	SW	AR	24.6 ± 0.1	7.1	220	0.127	+	25.3 ± 0.1	6.75	230	0.132	+
2	LW	AR	24.7 ± 0.1	6.9	210	0.121	+	24.7 ± 0.1	7.11	210	0.122	+
3	CC	AR	24.1 ± 0.1	7	200	0.118	+	24.2 ± 0.1	7.4	200	0.118	+
4	TB	AR	24.5 ± 0.1	7.1	220	0.131	+	23.5 ± 0.1	7.63	230	0.132	+
5	KB	AR	24.0 ± 0.1	6.9	160	0.094	−	23.4 ± 0.1	7.51	160	0.092	+
6	JP	LR	25.6 ± 0.2	7.2	280	0.165	+	24.1 ± 0.1	7.61	320	0.19	+
7	BB	LR	25.9 ± 0.2	7.2	290	0.17	+	24.4 ± 0.1	7.86	330	0.196	+
8	CG	LR	26.2 ± 0.2	7	370	0.221	+	25.2 ± 0.1	7.98	340	0.199	+
9	MB	LR	25.3 ± 0.2	7.4	380	0.225	+	26.2 ± 0.1	7.69	320	0.19	−
10	WLV	LR	27.4 ± 0.2	7.4	370	0.222	+	27.2 ± 0.1	7.52	310	0.184	−

^*a*^ SN, serial number.

^*b*^ L, location, SW, Stanmore Weir; LW, Luscombe Weir; CC, Cedar Creek, Chardons; TB, Tabragalba Bridge; KB, Kerry Bridge; JP, Josephville; BB, Bogan Bridge; CG, Cedar Grove; MB, Macleans Bridge, South Macleans; WLV, Wendt Park Logan Village.

^*c*^ R, river, AR, Albert River; LR, Logan River.

^*d*^ WT, water temperature.

^*e*^ C, conductivity @25^o^C us/cm.

^*f*^ S, salinity PSU.

^*g*^ + and −TV, presence/absence of toxigenic non-O1 and non-O139 *V*. *cholerae* from 1 mL broth culture from 6, 18, and 24 h of incubation.

By screening 80–90 colonies from each site, we were able to isolate 133 NOVC strains. Among these, 33 were toxigenic and 100 non-toxigenic NOVC isolates from various sites. From this collection of strains, 11 toxigenic and 11 non-toxigenic *V. cholerae* isolates were further characterized by WGS.

### Detection of biotype-specific genotypes and distribution of pathogenicity-associated virulence genes profiles and genomic islands

Among the 22 sequenced strains, 11 strains were cholera toxin positive and defined as toxigenic strains. While analyzing ctxB amino acid sequences, it was determined that seven toxigenic environmental *V. cholerae* non-O1 and non-O139 strains were genotype 2 with a point variation at the 35th position of the ctxB amino acid sequence as shown in Fig. 1 and Table 3. At position 35, Asparagine Asn (N) was replaced by Serine Ser (S) in environmental *V. cholerae* non-O1 and non-O139 strains from 2012 and in this study except for genotype 2 strains (KB1-31, KB1-39, KB1-40, KB1-4; Fig. 1). The Australian genotype 2 was based on the position of amino acids: His H (20), Gln Q (24), Asp D (28), His H (34), His H(39), Leu L(46), Lys K(55), and Thr T(68). In analyzing ctxA amino acid sequences, TB1-1, TB1-22, TB1-2, JP1-24, JP2-1, JP3-23, and MB3-24 showed similar ctxA amino acid sequences, whereas differences were found at D20N, H98Y, P121A, H149N, L153V, H158Y, G181A, and I190R compared to KB1- 31, KB1-39, KB1-40, and KB1-4 as shown in Data set 2.

**TABLE 3:** Diverse genotypes among NOVC strains from Australia

Strains	Biotype-specific genotypes^*a*^
JP1-24	*ctxB-*Aus*, rstR-*ET, and *tcpA-*CC
JP2-1	*ctxB-*Aus*, rstR-*ET, and *tcpA-*CC
JP3-23	*ctxB-*Aus*, rstR-*ET, and *tcpA-*ET
MB3-24	*ctxB-*Aus*, rstR-*ET, and *tcpA-*CC
TB1-22	*ctxB-*Aus*, rstR-*ET, and *tcpA-*ET
TB1-1	*ctxB-*Aus*, rstR-*ET, and *tcpA-*ET
TB1-2	*ctxB-*Aus*, rstR-*ET, and *tcpA-*ET
KB1-4	*ctxB-*Aus and *rstR-*CC
KB1-31	*ctxB-*Aus and *rstR-*CC
KB1-39	*ctxB-*Aus and *rstR-*CC
KB1-40	*ctxB-*Aus and *rstR-*CC

^*a*^ ctxB-Aus = Australia-specific ctxB, ET= El Tor, CC = classical.

Moreover, among the 11 toxigenic NOVC strains, three different biotype-specific genotypes with respect to the cholera toxin gene (*ctxB*), repetitive sequence transcriptional repressor (*rstR*), and toxin co-regulated pilus *tcpA* of classical (CC) and El Tor (ET) were reported as shown in Table 3. All 11 non-toxigenic NOVC were lacking the biotype-specific genotypes *ctxB*, *rstR,* and *tcpA*.

### Genomic characterization of environmental *V. cholerae* strains

**Fig 2:**
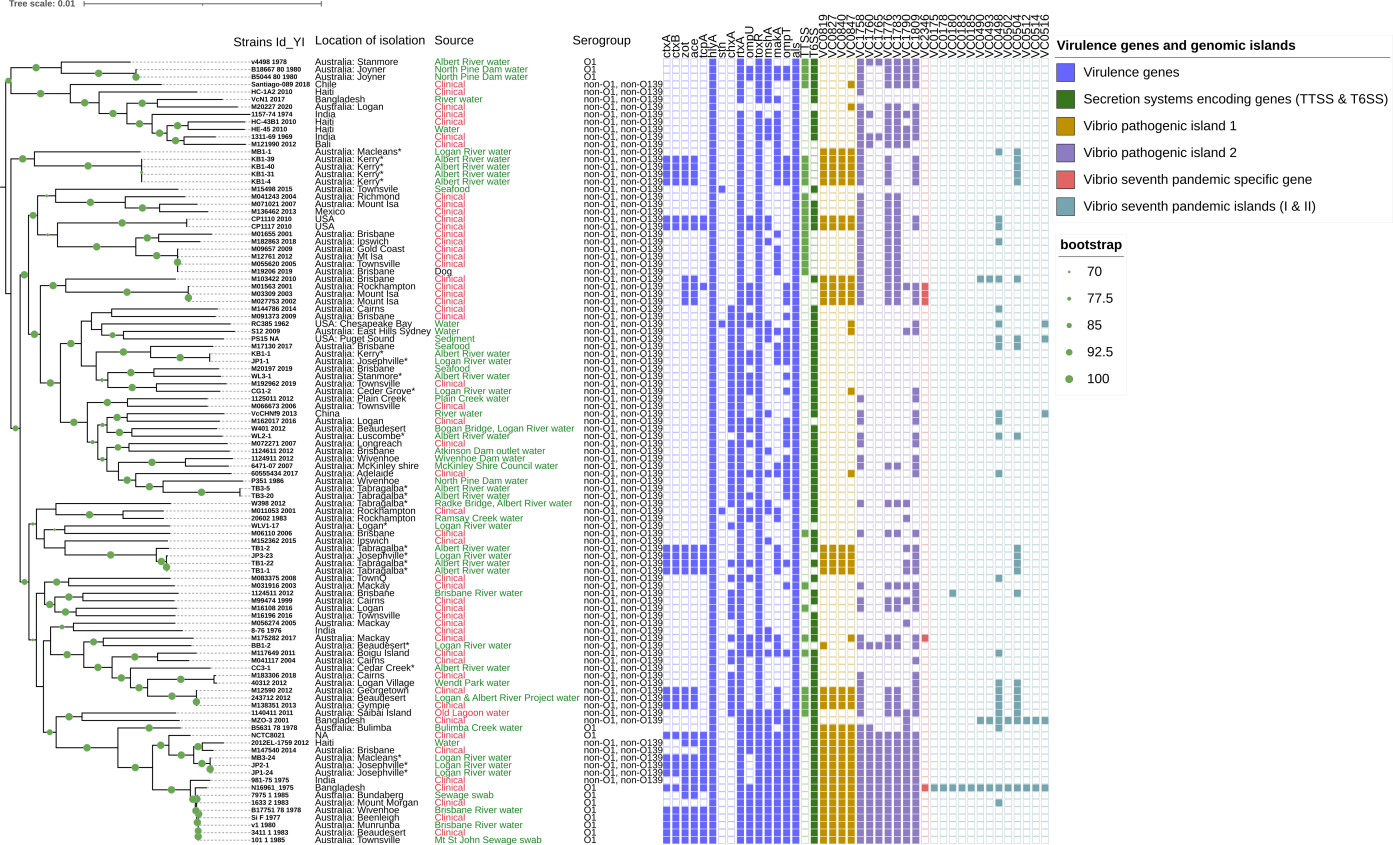
The maximum likelihood phylogenetic tree of 22 environmental non-O1 and non-O139 from this study and 83 previously sequenced *V. cholerae* strains based on SNP differences across the whole core genome, excluding likely recombination events and prophage regions. *V. cholerae* N16961 is used as a reference. Strain identities (IDs) with year of isolation, location of strains, virulence genes, Vibrio pathogenicity islands 1 & 2 (VPI-1 & 2), seventh pandemic-specific region, and Vibrio seventh pandemic islands I & II (VSP-I & II) profiles were generated using iTOL (https://itol.embl.de/) and represented as a colored box for presence and a white box for absence. Strains isolated (2021) and sequenced in this study were noted with an asterisk (*). Green color description represents the environmental and red represents the clinical source.

Among 105 *V*. *cholerae* strains used in this study for the maximum likelihood phylogenetic tree, 22 strains from this study, 63 strains from our previous studies, and 19 publicly available strains plus a reference strain (N16961) were used. More than 10 diverse clusters were observed with up to 19,000 SNP differences compared to the reference strain. Based on SNP analysis, toxigenic *V. cholerae* strains from Kerry, Albert River (KB1-40, KB1-4, KB1-39, and KB1-31) belonged to the same sub-cluster with 1–2 SNP differences as shown in Fig. 2 and Data set 3. Of note, high genomic diversity among non-O1 and non-O139 *V. cholerae* strains from the same location in the Logan and Albert Rivers, such as JP3-23 and TB3-5, TB3-20, showed 15,000 SNPs compared to JP1-24, JP2-1 & JP1-1, and TB1-1 & TB1-22 as shown in Fig. 2 and Data set 3. In addition, the non-toxigenic KB1-1 strain from the Albert River showed only 3 SNPs compared to the non-toxigenic JP1-1 strain from the Logan River, Fig. 2 and Data set 3. The remainder of the strains sequenced in this study clustered sporadically into different clusters as shown in Fig. 2.

All the sequenced strains were analyzed for the presence of virulence-associated genes (*ctxA*, *ctxB*, *zot*, *ace*, *ace*, *tcpA*, *hlyA*, *als*, *toxR*, *rtxA*, *ompU*, *ompT*, *mshA*, and *makA*) including type six secretion systems, vibrio pathogenicity islands VPI-1 & 2, and seventh pandemic islands (VSP-I & II) as shown in Fig. 2. Among 22 NOVC strains sequenced in this study, 50% (*n* = 11) harbored the CTX phage encoding genomic region, whereas only 43% (*n* = 7) of the toxigenic strains contained toxin co-regulated pilus (*tcpA*). All the toxigenic and non-toxigenic 22 *V*. *cholerae* strains showed the presence of hemolysin (*hlyA*), repeats in toxin gene (*rtxA*), and transcriptional activator (*toxR*) genes, whereas only 22% (*n* = 8) of the strains contained cholix toxin gene (*chxA*). None of the sequenced strains harbored the heat-stable enterotoxin (*stn*) gene. Only 11% (*n* = 4) of strains contained T3SS, whereas 59% strains contained T6SS. Interestingly, 54% (*n* = 12) of *V. cholerae* strains had complete Vibrio pathogenic island, VPI-1 and 18% (*n* = 4) contained VPI-2, with all lacking complete regions of seventh pandemic islands, VSP-I and VSP-II, Fig. 2.

### Antimicrobial resistance gene profile, class 1 integron, plasmids, and mobile genetic SXT elements

**Fig 3:**
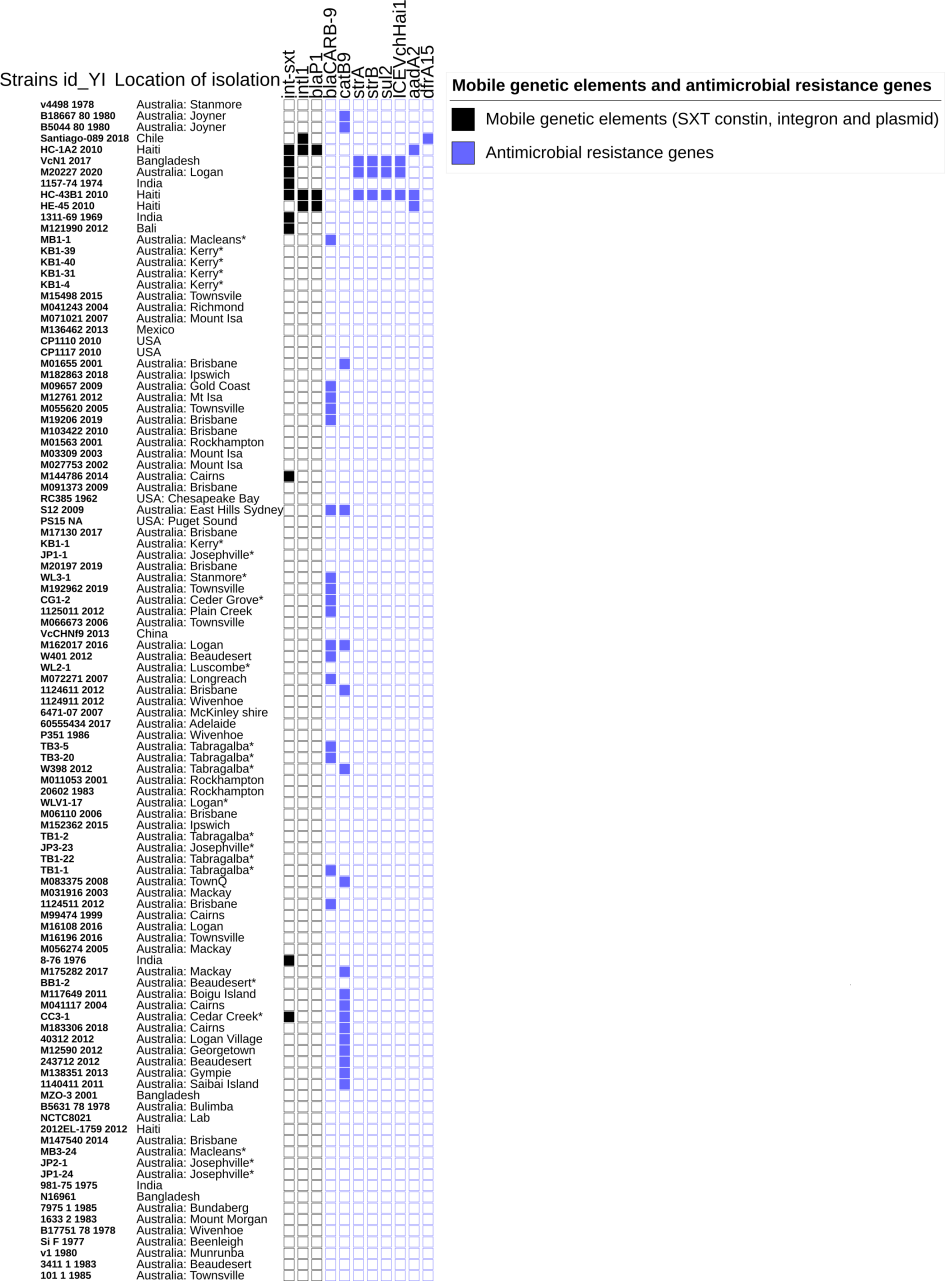
Antimicrobial resistance genes and mobile genetic element profiles of *V. cholerae* strain identifications with year of isolation (YI). Strains sequenced in this study are noted with an asterisk (*) at each isolation location. Colored boxes represent the presence and empty boxes represent the absence of genetic factors.

All the analyzed strains exhibited the same profile (no SNPs) of antimicrobial-resistance-associated genes: DNA gyrase subunit A, DNA topoisomerase IV subunit A, and DNA topoisomerase IV subunit B (*gyrA*, *parC,* and *parE*). Interestingly, in this study, environmental strain CC3-1 from Australia contained *int*SXT, mobile genetic element gene sequence and catB-9 as shown in Fig. 3. Notably, 27% (*n* = 6) strains contained *bla*CARB-9, a carbenicillinase that belongs to a family of cassette-encoded beta-lactamases. None of the environmental strains sequenced in this study contained plasmid-associated genes. The distribution of antibiotic resistance profiles for international *V. cholerae* strains was sporadic as shown in Fig. 3.

*Int*SXT, a mobile genetic element, is widely distributed in *Enterobacteriaceae* and commonly confers multidrug resistance. BLASTn (https://blast.ncbi.nlm.nih.gov/Blast.cgi) comparison of the CC3-1 *int*SXT sequence with the NCBI nucleotide collection showed similarities to other top six species with *int*SXT sequence from *Actinobacillus pleuropneumoniae* (Accession CP026009.1 and KX196444.1), *Shewanella* species (CP000503.1), *V. parahaemolyticus* (CP041202.1 and MN199028.1), *V. fluvialis* (JQ180502.1 and AB124846.1), *Proteus mirabilis* (CP021694.1), and *Alteromonas* species (CP018031.1 and CP065233.1). Interestingly, *int*SXT genes are being reported widely among enterobacteria and gamma proteobacteria families, and are not limited only to *Vibrio* sp. (*V. parahaemolyticus*, *V. fluvialis,* and *V. cholerae*). Regarding the multiple sequence alignment of *int*SXT amino acid sequences from this study (CC3-1), our previous study (M144786 and M20227), and compared to some of the closely related publicly available international strains conferring *int*SXT amino acid sequences, revealed genetic variations at several positions (123, 145, 284, 332, 333, and 334) and with high similarities within our study strains isolated at different time points (Table 4). Interestingly, our environmental strain CC3-1 had no *int*SXT amino acid sequence difference compared to the *P. mirabilis* strain AR_0155, United States (Table 4). Moreover, it was noteworthy to observe the variations in *int*SXT amino acid sequences among Queensland *V. cholerae*, *A. pleuropneumoniae* (App6), and *P. mirabilis* strains, whereas the rest of the international strains had similar sequences, although they are from different geographic regions.

**TABLE 4:** *int*SXT amino-acid sequence alignments of clinical and environmental *V. cholerae* strains^*a*^

				Amino acid position					
Strains ID	CO	YI	S	123	145	284	332	333	334
App6	China	2013	Clinical	Y	N	R	L	G	V
AR_0155	USA	NA	NA	Y	N	R	L	G	I
CC3-1	Australia	2021	Water	Y	N	R	L	G	I
M144786	Australia	2014	Clinical	Y	N	K	L	S	V
M20227	Australia	2020	Clinical	F	N	K	I	A	V
V21	Vietnam	2000	Clinical	Y	T	K	L	G	I
VC1786ICE	Haiti	2010	Clinical	Y	T	K	L	G	I
IDH_4268	India	2012	Clinical	Y	T	K	L	G	I
ICDC-2605	China	1998	Clinical	Y	T	K	L	G	I
2012EL-2176	Haiti	2012	Clinical	Y	T	K	L	G	I
MJ-1236	Bangladesh	1994	Clinical	Y	T	K	L	G	I

^*a*^ CO, country of origin; YI, year of isolation; S, source.

## DISCUSSION

*V. cholerae* remains a major public health problem, mainly in cholera-endemic areas of underdeveloped and developing Asian and African countries, where access to safe drinking water and proper sanitation are limited. In Australia, sporadic or imported cases of gastroenteritis caused by O1 and NOVC strains have been reported since the previous cholera outbreak in 1977–1987 (3, 19). *V. cholerae* is a habitat of aquatic environments and toxigenic *V. cholerae* O1 and non-O1, non-O139 isolates have been isolated previously in South-East Queensland river waterways during cholera outbreaks and thereafter. Therefore, it was important and interesting to conduct environmental surveillance of Logan and Albert Rivers to investigate the occurrence of *V. cholerae* strains and determine their virulence and antimicrobial resistance profiles.

Our previous study on clinical and environmental *V. cholera* non-O1 and non-O139 strains isolated from different parts of Queensland has indicated the occurrence of pathogenic potential *V. cholerae* in the environment causing illness among patients with limited information on their distribution and genotypic features. Moreover, some of the *V. cholerae* strains lacking classical virulence factors such as CT and TCP, yet capable of causing gastroenteritis among patients reported to healthcare centers, are of concern (3, 10). Clinical isolates with toxin genes are only further characterized in reference laboratories. Thus, together with our previous studies, we aimed to understand the virulence and antimicrobial profiles of Queensland, Australian *V. cholerae* non-O1 and non-O1 clinical and environmental strains. Currently, vaccination is one of the useful strategies to control cholera epidemics, notably for O1 and O139 serogroup strains. To date, no vaccine is available for non-O1 and non-O139 strains, which are pathogenic, have multiple drug resistance potential, and are capable of causing outbreaks. Thus, surveillance is important to understand any sources of outbreaks or disease clusters.

In this study, the detection of only NOVC strains may not reflect the actual context of *V. cholerae* in South-East Queensland river waterways. It is known that *V. cholerae* can be in a viable but non-cultural state*,* which reflects the possible occurrence of O1 serogroup strains in a non-culturable state while transferring its cholera toxin-producing CTX phage region to non-O1 and non-O139 strains (3). The genetic diversity among non-O1 and non-O139 strains was not sample collection site or river specific. Global warming, climate change, and the evolution of bacterial pathogens are of concern (18). Similar to other studies, our study also showed the high occurrence of *V. cholerae* in higher water temperature samples. In the first round of collection, the lowest salinity site Kerry Bridge (KB) had no toxigenic strains and even lower numbers of NOVC strains compared to other sites with higher salinity. However, we were not able to establish the correlation of salinity and occurrence of toxigenic, non-toxigenic, or the absence of *V. cholerae* strains in the second round of collection for the same sites. This is possibly due to substantial rainfall and a flood event occurring in the Albert and Logan Rivers at the time of sample collection.

In corroboration to our previous study, this study showed the existence of both classical and El Tor *rstR*, *tcpA,* and *ctxB* genotype 2 containing *V. cholerae* strains in the environment (3). However, the toxigenic clinical or environmental *V. cholerae* O1 strains from the 1980s in Queensland showed *rstR* of classical biotype, *tcpA* of El Tor biotype only, and *ctxB* genotype 2 (19). This might support the typical evolutionary process of *V. cholerae* in general over time despite their serogroups. Previously, it has been suggested that during the evolution of El Tor pandemic strains, traces of classical biotype features were reported for some time before their extinction. This supports the hypothesis of evolving Australian *ctxB* genotype 2 gene-containing strains in the Australian environment which are known as Australian Indigenous strains. Confirming our previous findings, this study of toxigenic NOVC strains (KB1-39, KB1-40, KB1-31, and KB1-4) isolated from Kerry, and Albert River lacked *tcpA* genes. Moreover, the point mutation at the 35th position of the ctxB amino acid sequences among recently isolated environmental strains revealed the uniqueness of current Queensland strains compared to other environmental toxigenic *V. cholerae* non-O1 and non-O139 strains in Queensland and internationally. It would be interesting to determine in the future if this point mutation is environmental NOVC strain specific or whether interchange can occur between clinical NOVC and O1/O139 *V. cholerae* strains. This could support the new genotype of ctxB amino acid sequences in Australia-specific genotype 2 *V*. *cholerae* strains. The role of CtxB in pathogenesis is to attach to the GM1 ganglioside receptor of the small intestine and assist CtxA to produce cholera toxin that affects/disrupts the chlorine ion transport system and causes excessive water release to balance the chlorine leading to diarrhea (31, 32). Thus, it would be interesting to understand how this mutation might play a role in the pathogenesis of cholera. In this study, we found that the majority of *V. cholerae* strains have acquired pathogenicity islands partially or completely, however, lacking the seventh pandemic islands. It has been suggested that the cumulative acquisition of pathogenicity islands may increase virulence and contribute to the spread and emergence of some enteropathogens (33). Several studies have reported the presence of T3SS and T6SS among clinical NOVC and its role in the pathogenesis of diarrheal illness. In this study, the prevalence of T3SS and T6SS encoding genomic islands of environmental *V. cholerae* among toxigenic and non-toxigenic strains might reflect the infectious state of the bacterium despite their toxigenicity profile. Thus, we support the hypothesis from a study from reference (34) on considering T3SS and T6SS genes as molecular risk markers for NOVC and may be useful in epidemiologic monitoring studies (34). It will be interesting to understand the expression of these virulence genes among toxigenic and non-toxigenic strains that play a role in the pathogenesis of the disease.

This study also supports the hypothesis on the interchangeability of mobile genetic elements at the inter- and intra-species levels. This implies the existence of mobile genetic elements in *V. cholerae* present in Queensland waterways and can easily be interchanged between other environmental strains. This interchange can potentially ease the acquisition of antimicrobial resistance genes at any time, thus the need for ongoing monitoring of Queensland waterways.

### Conclusion

Taken together, this combinatorial PCR and genomic study highlight the occurrence of *V. cholerae* strains with clinically important genetic signatures such as toxin genes and potential pathogenic markers, showing diverse antimicrobial resistance gene profiles and conferring mobile genetic element gene sequences which are of public health authorities concern. From a public health perspective, monitoring and characterization of *V. cholerae* in Queensland waterways is important to assist in rapidly identifying the source of any locally acquired disease clusters.

## Data Availability

Raw sequence files and associated metadata were deposited to NCBI with BioProject (ID) PRJNA954897.

## SUPPLEMENTAL MATERIAL

Dataset 1 aem.00472-23-s0001.xlsx List of strains used in this study.

Dataset 2 aem.00472-23-s0002.txt Multiple sequence alignment of ctxA amino-acid sequences.

Dataset 3 aem.00472-23-s0003.xlsx SNPs distance.

ASM does not own the copyrights to Supplemental Material that may be linked to, or accessed through, an article. The authors have granted ASM a non-exclusive, world-wide license to publish the Supplemental Material files. Please contact the corresponding author directly for reuse.
